# The global population of SARS-CoV-2 is composed of six major subtypes

**DOI:** 10.1038/s41598-020-74050-8

**Published:** 2020-10-26

**Authors:** Ivair José Morais, Richard Costa Polveiro, Gabriel Medeiros Souza, Daniel Inserra Bortolin, Flávio Tetsuo Sassaki, Alison Talis Martins Lima

**Affiliations:** 1 Departamento de Fitopatologia, Universidade de Brasília, Brasília, DF 70910-900 Brazil; 2 Departamento de Veterinária, Universidade Federal de Viçosa, Viçosa, MG 36570-900 Brazil; 3 Instituto de Ciências Agrárias, Universidade Federal de Uberlândia, Uberlândia, MG 38410-337 Brazil; 4 Instituto de Biotecnologia, Universidade Federal de Uberlândia, Monte Carmelo, MG 38500-000 Brazil

**Keywords:** Genome informatics, Computational biology and bioinformatics, Evolution, Population genetics, Genetic variation

## Abstract

The World Health Organization characterized COVID-19 as a pandemic in March 2020, the second pandemic of the twenty-first century. Expanding virus populations, such as that of SARS-CoV-2, accumulate a number of narrowly shared polymorphisms, imposing a confounding effect on traditional clustering methods. In this context, approaches that reduce the complexity of the sequence space occupied by the SARS-CoV-2 population are necessary for robust clustering. Here, we propose subdividing the global SARS-CoV-2 population into six well-defined subtypes and 10 poorly represented genotypes named tentative subtypes by focusing on the widely shared polymorphisms in nonstructural (*nsp*3, *nsp*4, *nsp*6, *nsp*12, *nsp*13 and *nsp*14) cistrons and structural (*spike* and *nucleocapsid*) and accessory (*ORF8*) genes. The six subtypes and the additional genotypes showed amino acid replacements that might have phenotypic implications. Notably, three mutations (one of them in the Spike protein) were responsible for the geographical segregation of subtypes. We hypothesize that the virus subtypes detected in this study are records of the early stages of SARS-CoV-2 diversification that were randomly sampled to compose the virus populations around the world. The genetic structure determined for the SARS-CoV-2 population provides substantial guidelines for maximizing the effectiveness of trials for testing candidate vaccines or drugs.

## Introduction

In December 2019, a local pneumonia outbreak of initially unknown aetiology was detected in Wuhan (Hubei, China) and quickly determined to be caused by a novel coronavirus^1^, named severe acute respiratory syndrome coronavirus 2 (SARS-CoV-2)^2^, with the disease referred to as COVID-19^3^. SARS-CoV-2 belongs to family *Coronaviridae*, genus *Betacoronavirus*, which comprises enveloped, positive-stranded RNA viruses of vertebrates^2^. Two-thirds of SARS-CoV genomes are covered by ORF1ab, which encodes a large polypeptide that is cleaved into 16 nonstructural proteins (NSPs) involved in replication-transcription in vesicles from endoplasmic reticulum (ER)-derived membranes^4,5^. The last third of the virus genome encodes four essential structural proteins, namely, Spike (S), Envelope (E), Membrane (M), and Nucleocapsid (N), and several accessory proteins that interfere with the host innate immune response^6^.

Populations of RNA viruses evolve rapidly due to their large sizes, short generation times, and high mutation rates, the last of which is a consequence of RNA-dependent RNA polymerase (RdRP), which lacks proofreading activity^7^. In fact, virus populations are composed of a broad spectrum of closely related genetic variants resembling one or more master sequences^8–10^. Mutation rates inferred for SARS-CoVs are considered moderate^11,12^ due to independent proofreading activity^13^. However, the large SARS-CoV genomes (from 27 to 31 kb)^14^ allow efficient exploration of the sequence space^15^. To better understand the diversification of SARS-CoV-2 genomes during the pandemic (from December 2019 to March 25, 2020), we applied a simple but robust approach to reduce the complexity of the sequence space occupied by the virus population by detecting its widely shared polymorphisms.

**Figure 1:**
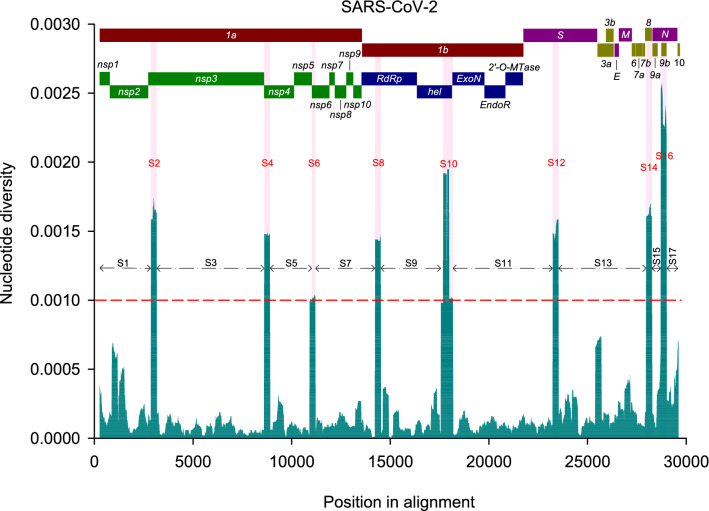
Mean pairwise number of nucleotide differences per site (nucleotide diversity, π) calculated using a sliding window of 300 nucleotides across the multiple sequence alignment for full-length genomes of SARS-CoV-2. The red dashed line at π = 0.001 represents an arbitrary threshold used to subdivide the segments (S) with higher (S2, 4, 6, 8, 10, 12, 14 and 16) and lower (S1, 3, 5, 7, 9, 11, 13,15 and 17) levels of genetic variation. The SARS-CoV-2 genome organization is represented on top of the plot.

A total of 767 SARS-CoV-2 genomes with high sequencing coverage obtained from GISAID (https://www.gisaid.org/) and GenBank were clustered into 593 haplotypes (Table S1). We conducted a fine-scale sequence variation analysis of the 593 genome-containing alignment by calculating nucleotide diversity (π) using sliding window and step sizes of 300 and 20 nucleotides, respectively (multiple sequence alignments generated in this study are available from the authors upon request). Such an approach allows the identification of genomic regions with increased genetic variation from polymorphic sites harbouring two or more distinct nucleotide bases. Noticeably, one or more large clusters of closely related sequences, when analysed by this approach, show locally increased nucleotide diversity. We observed contrasting distributions of genetic variation across the full-length genomes of SARS-CoV-2 (Fig. 1), with eight segments (S) showing increased genetic variation, arbitrarily defined as nucleotide (nt) segments with π ≥ 0.001. Seven out of eight segments were approximately 280 nucleotides (nt) in length, corresponding approximately to the size of a single sliding window, except S10, whose length was equivalent to two sliding windows (600 nt). To further investigate the diversification of segments with contrasting degrees of genetic variability, we constructed maximum likelihood (ML) phylogenetic trees and analysed the diversification patterns of eight segments with higher genetic variation (S2, 4, 6, 8, 10, 12, 14 and 16) and nine with lower genetic variation (S1, 3, 5, 7, 9, 11, 13, 15 and 17).

Although the data set was composed of hundreds of SARS-CoV-2 genomes sampled from around the world, in the S2-based tree, we observed two clusters (Fig. S1a). Notably, each cluster was composed of very closely related, if not identical, sequences. Therefore, the increased degree of genetic variation at S2 was a result of inter-cluster sequence comparisons. Similar results were obtained for the other seven ML trees based on segments with increased genetic variation (Fig. S1b–h). In contrast, the ML trees based on segments with lower genetic variation did not show a consistent number of well-defined clusters (Fig. S2).

We mapped the polymorphic sites in segments with increased genetic variation responsible for the segregation of ML trees into two well-defined clusters (Table 1). Only a few (from one to three) nt positions with polymorphisms shared by a number of SARS-CoV-2 genomes could be identified within each segment with increased genetic variation. These polymorphisms were henceforth referred to as ‘widely shared polymorphisms’ (WSPs), while the remaining nt positions in virus genomes were designated as ‘non-widely shared polymorphisms’ (nWSPs).

**Table 1:** Characterization of the WSPs detected in genomes of SARS-CoV-2.

Segment ID	Segment position^a^ (begin–end)	WSPs^b^	nt mutation (# isolates)	Position in the codon	#codon	Amino acid
S2	2899–3179	*nsp3-*[3,037]	**U** (184)/**C** (409)	Third	106	Phenylalanine/Phenylalanine
S4	8639–8919	*nsp4-*[8,782]	**U** (183)/**C** (410)	Third	76	Serine/Serine
S6	10,959–11,219	*nsp6-*[11,083]	**C** (1)/**U** (99)/**G** (493)	Third	37	Phenylalanine/Phenylalanine/Leucine
S8	14,259–14,539	*nsp12-*[14,408]	**U** (184)/**C** (409)	Second	323	Leucine/Proline
S10	17,600–18,200	*nsp13-*[17,747]	**U** (101)/**C** (492)	Second	504	Leucine/Proline
		*nsp13-*[17,858]	**G** (101)/**A** (492)	Second	541	Cysteine/Tyrosine
		*nsp14-*[18,060]	**U** (105)/**C** (488)	Third	7	Leucine/Leucine
S12	23,270–23,550	*S-*[23,403]	**G** (185)/**A** (408)	Second	614	Glycine/Aspartate
S14	28,004–28,285	*ORF8-*[28,144]	**C** (184)/**U** (409)	Second	84	Serine/Leucine
S16	28,745–29,025	*N-*[28,881]	**A** (60)/**G** (533)	Second	203	Lysine/Arginine
		*N-*[28,882]	**A** (60)/**G** (533)	Third		
		*N-*[28,883]	**C** (60)/**G** (533)	First	204	Glycine/Arginine

^a^Relative to the multiple sequence alignment constructed for full-length genomes.

^b^Widely shared polymorphism (WSP) positions are relative to the reference genome (GISAID accession ID: EPI_ISL_402124).

**Figure 2:**
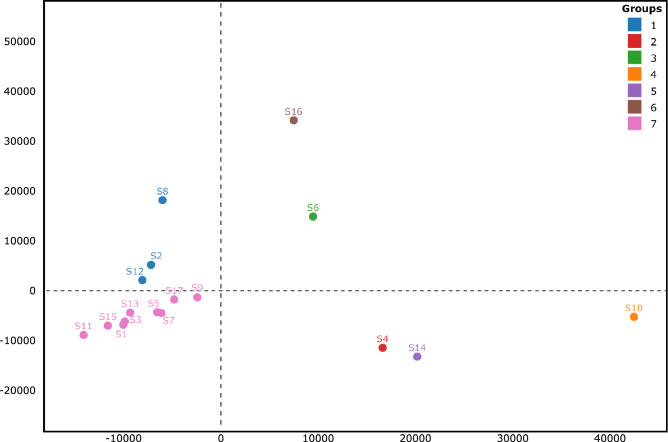
Multidimensional scaling (MDS) visualization of tree distances based on the Kendall-Colijn metric (λ = 0). The seventeen ML trees (each with 593 tips) are represented as dots, and groups of trees showing similar topologies are indicated by the same colour. The WSP-containing segment-based trees formed six groups: the first group comprised S2, S8 and S12 (indicated in blue), while the other five were represented by single trees (groups 2–6 indicated in red, green, orange, purple and brown, respectively). All nWSP-containing segment-based ML trees formed a single group, indicated in pink.

We compared the topologies of the seventeen ML trees (Figs. S1 and S2) by computing their pairwise distances followed by a multivariate analysis to group similar trees (Fig. 2). The seventeen trees were subdivided into seven groups, with the largest including nWSP-containing segment-based trees (S1, 3, 5, 7, 9, 11, 13, 15 and 17; Fig. 2, Group 7). Given the low genetic variation in these segments, the resulting trees were poorly resolved, suggesting that such regions represent a wide mutant spectrum of narrowly shared polymorphisms. It is important to note that there are minor clusters in nWSP-containing segment-based ML trees, e.g., in those for S1, S13 and S17. This is a consequence of our conservative threshold, as we focused on segments with π ≥ 0.001. S1, S13 and S17 also show locally increased genetic variation with π values higher than 0.0005 but lower than 0.001, for example, stretches 916–1196, 1436–1536 (within S1), 25,430–25,720 (S13), 29,565–29,637 (S17) (Fig. 1).

The S2-, S8- and S12-based ML trees (Fig. 2, Group 1) were considerably congruent, and the nucleotides at their WSPs tended to co-segregate (UUG or CCA, Table 1), which resulted in two major subtypes of SARS-CoV-2 (Figs. S1a, 1d and 1f). Reciprocally, the incongruency among the S4-, 6-, 10-, 14- and 16-based trees (Fig. 2, Groups 2–6) suggests the segregation of nucleotides at their WSPs, which increases the possible combinations of virus genotypes.

Therefore, our approach reduced the complexity of the sequence space occupied by the SARS-CoV-2 genomes and provided a robust clustering solution based on the combination of 12 WSPs (Table 1) to barcode the major viral genotypes spread worldwide (Table 2 and Table S2). The global population of SARS-CoV-2 is structured into six major subtypes (I–VI), comprising 578 of 593 (approximately 97.5%) isolates analysed in this study. Subtype I (N = 132) was represented by the combination of the most frequent nucleotides at all WSPs, i.e., the canonical genotype CCGCCACAUGGG. The SARS-CoV-2 reference genome (GISAID accession ID: EPI_ISL_402124, GenBank accession: MN908947) is a representative member of this subtype. Subtype IV (N = 91) was represented by the combination of the most frequent nucleotides at eleven of 12 WSPs (**CC**U**CCACAUGGG**; the most frequent nucleotides at each WSP are highlighted in bold and underlined). Subtypes V (N = 74, **C**U**GCCACA**C**GGG**), II (N = 122, U**CG**U**CAC**G**UGGG**), III (N = 101, **C**U**GC**UGU**A**C**GGG**) and VI (N = 58, U**CG**U**CAC**G**U**AAC) were represented by the combination of the most frequent nucleotides at ten, nine, seven and six of 12 WSPs, respectively.

**Table 2:**
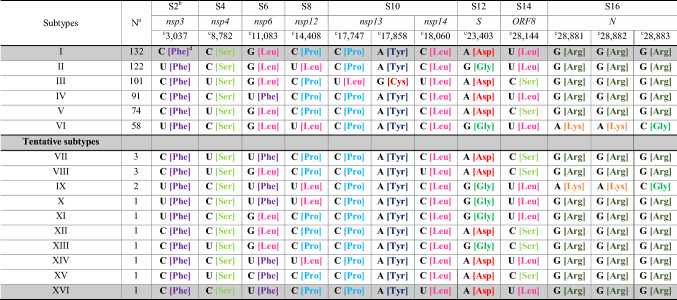
Unique genotypes of SARS-CoV-2 based on 12 WSPs and their associated amino acid replacements.

^a^Sample size. ^b^Segment containing the WSP. ^c^Nucleotide position relative to the reference genome (GISAID accession ID: EPI_ISL_402124). ^d^Nucleotide base and the encoded amino acid residue.

**Figure 3:**
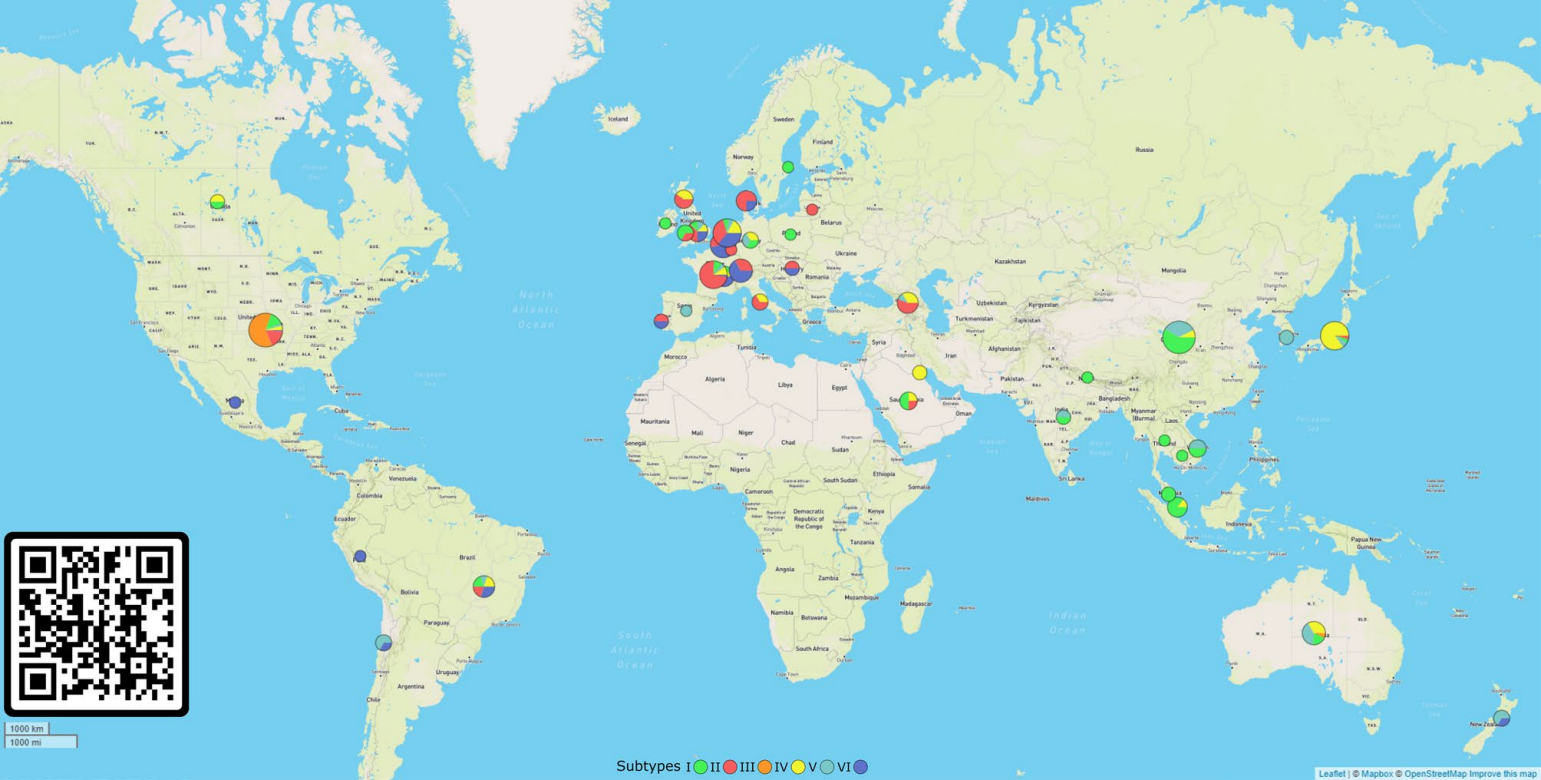
Geographical distribution of six subtypes of SARS-CoV-2 around the world. The genomic data set comprised isolates sampled from 40 distinct countries from December 24, 2019 to March 20, 2020. The pie charts show the proportion of each subtype of SARS-CoV-2 according to a colour key in the figure bottom. For more detailed information on virus spread, a dynamic map is available at https://microreact.org/project/f25A3jAvE5TjzxAf38UCEq (accessible via the QR code in the bottom left corner of the map).

**Figure 4:**
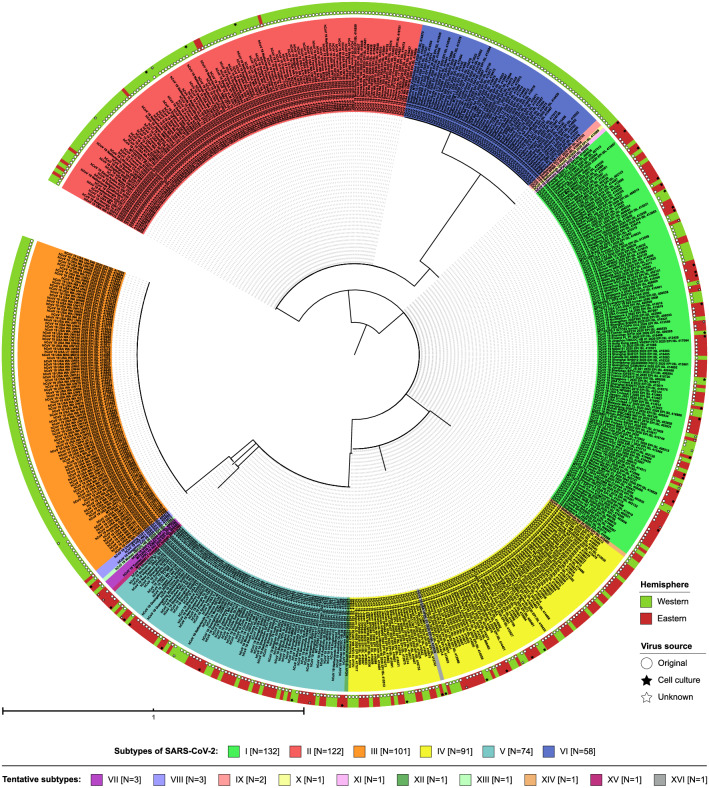
Maximum likelihood phylogenetic tree based on 12 WSPs detected across the SARS-CoV-2 genomes. The background colour of the tips indicates the subtype (I–VI) or tentative subtype (VII–XVI). An outer strip indicates the geographic origin (Western or Eastern Hemisphere) and whether each isolate was subjected to intermediate cell culture passages before genome sequencing.

It is important to note the intrinsic wide geographical coverage of these subtypes, since they were sampled from distinct countries or even continents, which describes the viral spread at a global scale (Fig. 3). A dynamic map of the spatial–temporal spread of isolates of the six subtypes of SARS-CoV-2 is available as a Microreact project (https://microreact.org/project/f25A3jAvE5TjzxAf38UCEq). Another important feature is that they are predominantly composed of genomes sequenced from original samples, minimizing any mutational bias due to in vitro virus replication (Fig. 4). Studies on the mutational dynamics of SARS-CoV-2 in cell culture have not been conducted thus far; however, previous studies on the mutational dynamics of SARS-CoV indicated a negligible mutation frequency after five serial Vero-E6 cell passages^16^.

Ten additional viral genotypes were poorly represented and, therefore, referred to as tentative subtypes (Table 2). This category of tentative subtypes would be useful due to the continuous addition of genomes to public databases, where more representative members might be sampled from a wider geographical context. This conservative proposal would keep the inclusive nature of our clustering method, being able to incorporate a large fraction of the SARS-CoV-2 genetic variation at a global scale.

The WSP-based phylogenetic tree depicting all 593 SARS-CoV-2 haplotypes (Fig. 4) showed some geographical structure with two clusters: a smaller cluster comprising isolates mostly sampled from the Western Hemisphere (Subtypes II and VI; tentative Subtypes IX, X and XI) and a larger cluster comprising isolates sampled from the Western and Eastern Hemispheres (Subtypes I, III, IV and V; tentative Subtypes VII, VIII, XII–XVI). The co-segregation of nucleotides at WSPs *nsp3*-[3,037], *nsp12*-[14,408] and *S*-[23,403] (Fig. 2) was responsible for the geographical structure in our ML tree (Fig. 4). The mutation *nsp3*^U3,037C^ led to synonymous codons for phenylalanine in haplotypes from both clades, while the mutation *nsp12*^U14,408C^ was non-synonymous, leading to leucine and proline in haplotypes from Western and Western/Eastern Hemisphere clades, respectively (Table 2). The S^G23,403A^ mutation led to non-synonymous codons for glycine and aspartate in haplotypes from Western and Western/Eastern Hemisphere clades, respectively. Together, these results suggest the predominance of NSP12^L323^ and Spike^G614^ in the Western Hemisphere.

The WSP-based tree (Fig. 4) included only 12 nt sites, which represented 0.04% of the full-length multiple sequence alignment (29,412 nt after trimming the poor-quality 5′ and 3′ untranslated regions). Notably, the topologies of the WSP-based tree and the tree based on full-length genomes (Fig. S3) were highly similar, indicating a parsimonious approach for directly identifying the most informative sites in these viral genomes.

Virus barcoding and phylogenetic approaches have been conducted for SARS-CoV^17^ and Middle East respiratory syndrome coronavirus (MERS-CoV)^18^, respectively. Due to the limited geographical coverage of the MERS-CoV epidemic, a study focusing on a region of Saudi Arabia tracked and distinguished two main genotypes of circulating MERS-CoV. On the other hand, 174 polymorphic loci in 101 complete genomes and 44 partial sequences of SARS-CoV allowed to further subdivide its population into two previously identified genotypes (named C and T)^19^ and an additional eight “subgenotypes” (C1–C4 and T1–T4) due to 10 special loci or informative sites^17^. Interestingly, genotype C was compatible with the virus isolated during regional transmission and the early and intermediate phases of the SARS-CoV epidemic in the years 2003–2004, while genotype T was compatible with a viral type of international transmission from the late stage of virus spread^20,21^. These results are similar to those of our study, which subdivided the genomes into major lineages of SARS-CoV-2. Therefore, the epidemic subdivisions of SARS-CoV and SARS-CoV-2 are apparent and similar because of possible viral adaptation as the virus spreads throughout the world.

Studies have been conducted to discriminate closely related bacterial taxa using Shannon entropy as a metric of sequence information content^22^. A similar approach was recently applied to track the geographical and temporal dynamics of SARS-CoV-2^23^. In the latter study, analogous to our 12 WSP-based genotypes, 17 informative subtype markers (ISMs) were employed and revealed nine subtypes as the most represented in the overall virus population. All 12 WSPs detected in our study were identified as ISM sites, which indicates that nucleotide diversity is also an informative metric with which to search for subtype signatures. Notably, the ISMs were proposed to track the virus spread within and between countries and/or continents, while our WSP-based approach was focused on highlighting the potential biological implications of such founding mutations since nine of 12 lead to non-synonymous replacements at the protein level.

It is important to note that the mutations at WSPs *nsp13*-[17,747 and 17,858] were responsible for the segregation of Subtype III, that is, were redundant, and only one would be sufficient to reproduce the segregation into six major and 10 tentative subtypes. As a consequence, the set of WSPs necessary for subdivision identical to that shown in this study might be reduced to 11 nucleotide bases. However, we kept such informative sites according to the proposed threshold in our fine-scale genetic variation analyses, and they might be useful in a scenario where novel subtypes (partially similar to the genotype of Subtype III) are included due to the combined efforts of many countries to sequence thousands of SARS-CoV-2 genomes.

We hypothesize that our clustering method for the SARS-CoV-2 population could involve a biological context to some extent. The WSP *nsp6-*[11,083] in Subtype IV of SARS-CoV-2 led to phenylalanine at aa residue #37 of the protein and leucine in five other subtypes (Table 2). NSP6 is an integral membrane protein that interferes with autophagosome formation during SARS-CoV infection. Additionally, in yeast two-hybrid experiments^24^, NSP6 has been shown to interact with NSP3. Some evidence demonstrates that NSP6 protein limits the expansion of autophagosomes or, alternatively, might remove host proteins involved in the inhibition of viral replication by activating autophagy from the ER^25^.

The WSP *nsp12-*[14,408] resulted in proline in four subtypes of SARS-CoV-2 and leucine in two other subtypes at aa residue #323 of the NSP12 (RNA-dependent RNA polymerase, RdRP) protein. This WSP is located at the interface domain of RdRP of SARS-CoV-2, which is responsible for the connection between the nidovirus RdRP-associated nucleotidyltransferase domain (NiRAN) and the “right hand” polymerase domain^26^. The S protein mediates viral entry into host cells by first binding to a receptor, angiotensin-converting enzyme 2 (ACE2), through the receptor-binding domain (RBD) in the S1 subunit and then fusing the viral and host membranes through the S2 subunit^27–30^. Sites of glycosylation are important for S protein folding^31^, affecting priming by host proteases^32^ and might modulate antibody recognition^33,34^. The WSP *S-*[23,403] resulted in glycine and aspartate at aa residue #614 of the S protein in two and four subtypes of SARS-CoV-2, respectively. The replacement was mapped to the intermediate region between the S1 and S2 subunits. This WSP is near a glycosylation site (N616CT)^35^.

The WSP *ORF8-*[28,144] involved a non-synonymous mutation at codon #84 encoding leucine and serine in four and two subtypes, respectively. SARS-CoV *ORF8* encodes an ER-associated protein that induces Activation of Transcription Factor 6 (ATF6), which is an ER stress-regulated transcription factor that stimulates the production of chaperones^36^. The ORF8 protein has also been demonstrated to induce apoptosis^37^. In the SARS epidemic, *ORF8* was targeted by a number of mutations and recombination events during transmission from non-human animals to humans^38^.

Three consecutive WSPs mapped in the *N* gene led to two amino acid replacements at residues #203 and #204. The multifunctional N protein is composed of three domains^39^, two of which are structurally independent: the N-terminal domain (NTD) and the C-terminal domain (CTD). Both amino acid replacements were mapped to an intermediary domain referred to as the linker region (LKR), a positively charged serine-arginine-rich region. As an intrinsically disordered region (IDR), it allows the independent folding of the NTD and CTD^40^ and is also functionally implicated in RNA binding activity^39^. Key determinants of the interaction between the N and NSP3 proteins were also mapped to the LKR^41^. The SARS-CoV N protein is also responsible for an antigenic response in humans predominantly involving immunoglobulin G^42^. Although the host biological factors involved in the response to SARS-CoV-2 infection are still poorly known, the existence of distinct virus subtypes, all of them exhibiting amino acid replacements, could affect important aspects of COVID-19.

We hypothesized that in the early stages of the SARS-CoV-2 epidemic, due to rapid virus population expansion, a number of genetic variants might have arisen, followed by their spread to other countries and continents. We argue that the virus subtypes and their associated WSPs detected in this study could serve as records of diversification in these early stages of the epidemic after transmission from non-human animals to humans. After virus introduction to a given geographic region, a number of unique or narrowly shared mutations accumulate; however, most of them reduce fitness and are removed by purifying selection on a medium- to long-term evolutionary scale, tending to decrease genetic variability^8^.

Therefore, we propose classifying SARS-CoV-2 into at least six distinct subtypes accounting for more than 97% of the isolates sampled from around the world. Such classification might guide the validation of candidate vaccines or drugs for the widest range of virus subtypes. In this context, our clustering solution provides a robust approach for effectively reducing the complexity of the mutant spectrum involving closely related SARS-CoV-2 genomes and a focus on WSPs. Additionally, through exhaustive sequencing, it would be possible to change the tentative status of the ten genotypes described in this study or even identify novel virus subtypes and follow the evolutionary dynamics of the SARS-CoV-2 population during the adaptation process imposed by the human host.

## Methods

A total of 1,137 full-length genomes of SARS-CoV-2 were obtained from GenBank^43^ and GISAID^44^ (Table S1) on March 25, 2020, and comprised virus isolates sampled from December 24, 2019, to March 20, 2020. Only genomes with high sequencing coverage, intact ORFs (no frameshifts, except that of the *nsp*12 cistron) and no indeterminate nucleotide bases (indicated by ‘N’s or ambiguous codes), totalling 767 high-quality full-length sequences, were effectively analysed in this study. We wish to acknowledge all researchers who deposited the SARS-CoV-2 genomes in the GISAID and/or GenBank database.

The genomic data set was aligned using MAFFT-FFT-NS-2^45^. The calculation of the average number of nucleotide differences per site (nucleotide diversity, π) was conducted in DnaSP v.6^46^ using sliding window and step sizes of 300 and 20 nucleotides, respectively. Sites with gap alignment were not considered in the analysis.

Maximum likelihood (ML) phylogenetic trees were constructed using RAxML^47^ under the general time-reversible with gamma distribution (GTRGAMMA) nucleotide substitution model. The branch support for ML trees based on 300 nucleotides and larger segments was assessed with 1000 and 5000 bootstrap replicates, respectively. The ML tree for full-length genomes was based on a multiple alignment whose 5′ and 3′ untranslated regions were trimmed. ML trees were used in this study essentially as a clustering method due to the weak phylogenetic signal in the data set. All phylogenetic trees were edited using iTOL^48^. To assess the similarity among ML-tree topologies, we computed all possible pairwise distances by applying the Kendall–Colijn metric^49^, followed by principal coordinate analysis (PCoA), using the package treespace^50^ in R^51^.

The detection of polymorphic sites was conducted using PAUP* v. 4.0^52^ and MEGA X^53^. The sites responsible for the segregation of the isolates into two clusters in the ML trees were referred to as “widely shared polymorphisms” (WSPs), while the remaining nt positions in the virus genomes were designated as “non-widely shared polymorphisms” (nWSPs). The WSP positions were relative to the reference genome (GISAID accession ID: EPI_ISL_402124).

A Microreact project v70.0.0 was created for the metadata in a dynamic user interface^54^. Interactive visualization makes it possible to track virus sampling from a spatial–temporal perspective. A QR code for the interactive map was generated using the R package qrcode^55^.

## Supplementary information

Supplementary Figure 1a.

Supplementary Figure 1b.

Supplementary Figure 1c.

Supplementary Figure 1d.

Supplementary Figure 1e.

Supplementary Figure 1f.

Supplementary Figure 1g.

Supplementary Figure 1h.

Supplementary Figure 2a.

Supplementary Figure 2b.

Supplementary Figure 2c.

Supplementary Figure 2d.

Supplementary Figure 2e.

Supplementary Figure 2f.

Supplementary Figure 2g.

Supplementary Figure 2h.

Supplementary Figure 2i.

Supplementary Figure 3.

Supplementary Table 1.

Supplementary Table 2.

Supplementary Information.

## Data Availability

The multiple sequence alignments and ML phylogenetic trees generated in this study are available from the authors upon request. The Microreact project is available at https://microreact.org/project/f25A3jAvE5TjzxAf38UCEq.
