# Supplementary material for: The global population of SARS-CoV-2 is composed of six major subtypes
Source: Sci Rep. 2020 Oct 26;10:18289. doi: 10.1038/s41598-020-74050-8 (PMC7588421; doi:10.1038/s41598-020-74050-8)
Supplement: Supplementary file 21 — Supplementary Information. [file 41598_2020_74050_MOESM21_ESM.pdf]

Supplementary Material Description

The global population of SARS-CoV-2 is composed of six major subtypes

Ivair José Morais Júnior, Richard Costa Polveiro, Gabriel Medeiros Souza, Daniel Inserra Bortolin, Flávio Tetsuo Sassaki, Alison Talis Martins Lima

The Supplementary Material for this manuscript comprises:

1. Supplementary Figures S1, S2 and S3 are refer to maximum likelihood (ML) phylogenetic trees for segments with increased and lower genetic variation content and full-length genomes of SARS-CoV-2, respectively. Legends are provided along the figures.
2. Supplementary Tables S1 and S2 provided as spreadsheets in xlsx format and refer to the SARS-CoV-2 genomes used in this manuscript and their genotypes inferred based on 12 widely shared polymorphisms, respectively. Legends for the Supplementary Tables S1 and S2 are provided below in this document.

Supplementary Table S1 | Genomes of SARS-CoV-2 used in this study.

Genomes of SARS-CoV-2 were retrieved from GISAID[1] and GenBank[2] on 25 March, 2020. We applied a conservative filter for high quality sequences sampled from humans, intact ORFs (no frameshifts, except that of the *nsp*12 cistron) and no indeterminate nucleotide bases (indicated by 'N's or ambiguous codes). After filtering for high quality sequences, a total of 767 SARS-CoV-2 genomes were submitted to haplotype detection analysis in DnaSP v.6[3] and a single representative of each haplotype was used in study. Additional sequences from the same haplotype are indicated in red. The haplotypes 4, 37, 119 and 443 (31, 9, 7 and 6 genomes each, respectively) and 57, 168, 247, 318 (5 genomes, each) were the most represented in our data set. Therefore, after removing the redundant sequences, the data set totalled 593 unique SARS-CoV-2 genomes. We wish to acknowledge all researchers who deposited the SARS-CoV-2 genomes in GISAID and/or GenBank databases.

Supplementary Table S2 | Genotypes of 593 SARS-CoV-2 genomes based on 12 widely shared polymorphisms (WSPs) detected in this study.

For each virus isolate is shown the nucleotide at the WSPs: *nsp*3-[3,037], *nsp*4-[8,782], *nsp*6-[11,083], *nsp*12-[14,408], *nsp*13-[17,747], *nsp*13-[17,858], *nsp*14-[18,060], *S*-[23,403], *ORF8*-[28,144], *N*-[28,881], *N*-[28,882] and *N*-[28,883]. Nucleotide bases A, C, G and U are indicated by colored cells in green, blue, purple and red.

References

1. Shu, Y. & McCauley, J. GISAID: Global initiative on sharing all influenza data – from vision to reality. *Eurosurveillance* **22**, 2–4 (2017).
2. Sayers, E. W. *et al*. GenBank. *Nucleic Acids Res.* **47**, D94–D99 (2019).
3. Rozas, J. *et al*. DnaSP 6: DNA Sequence Polymorphism Analysis of Large Data Sets. *Mol. Biol. Evol.* **34**, 3299–3302 (2017).
